# Genome downsizing, physiological novelty, and the global dominance of flowering plants

**DOI:** 10.1371/journal.pbio.2003706

**Published:** 2018-01-11

**Authors:** Kevin A. Simonin, Adam B. Roddy

**Affiliations:** 1 Department of Biology, San Francisco State University, San Francisco, California, United States of America; 2 School of Forestry and Environmental Studies, Yale University, New Haven, Connecticut, United States of America; University of Cambridge, United Kingdom of Great Britain and Northern Ireland

## Abstract

The abrupt origin and rapid diversification of the flowering plants during the Cretaceous has long been considered an “abominable mystery.” While the cause of their high diversity has been attributed largely to coevolution with pollinators and herbivores, their ability to outcompete the previously dominant ferns and gymnosperms has been the subject of many hypotheses. Common among these is that the angiosperms alone developed leaves with smaller, more numerous stomata and more highly branching venation networks that enable higher rates of transpiration, photosynthesis, and growth. Yet, how angiosperms pack their leaves with smaller, more abundant stomata and more veins is unknown but linked—we show—to simple biophysical constraints on cell size. Only angiosperm lineages underwent rapid genome downsizing during the early Cretaceous period, which facilitated the reductions in cell size necessary to pack more veins and stomata into their leaves, effectively bringing actual primary productivity closer to its maximum potential. Thus, the angiosperms' heightened competitive abilities are due in no small part to genome downsizing.

## Author summary

The angiosperms, commonly referred to as the flowering plants, are the dominant plants in most terrestrial ecosystems, but how they came to be so successful is considered one of the most profound mysteries in evolutionary biology. Prevailing hypotheses have suggested that the angiosperms rose to dominance through an increase in their maximum potential photosynthesis and whole-plant carbon gain, allowing them to outcompete the ferns and gymnosperms that had previously dominated terrestrial ecosystems. Using a combination of anatomy, cytology, and modelling of liquid water transport and CO_2_ exchange between leaves and the atmosphere, we now provide strong evidence that the success and rapid spread of flowering plants around the world was the result of genome downsizing. Smaller genomes permit the construction of smaller cells that allow for greater CO_2_ uptake and photosynthetic carbon gain. Genome downsizing occurred only among the angiosperms, and we propose that it was a necessary prerequisite for rapid growth rates among land plants.

## Introduction

The flowering plants are highly competitive in almost every terrestrial ecosystem, and their rapid rise during the early Cretaceous period irrevocably altered terrestrial primary productivity and global climate [1–3]. Terrestrial primary productivity is ultimately determined by the photosynthetic capacity of leaves. The primary enzyme in photosynthesis, rubisco, functions poorly when CO_2_ is limiting, which requires leaf intercellular CO_2_ concentrations (*c*_i_) to be maintained within a narrow range [4] through adjustments in leaf surface conductance to CO_2_ and water vapor. This surface conductance is one of the greatest biophysical limitations on photosynthetic rates across all terrestrial plants [5,6]. In order for CO_2_ to diffuse from the atmosphere into the leaf, the wet internal surfaces of leaves must be exposed to the dry ambient atmosphere, which can cause leaf desiccation and prevent further CO_2_ uptake. As a consequence, increasing leaf surface conductance to CO_2_ also requires increasing rates of leaf water transport in order to avoid desiccation [7].

Both theory and empirical data suggest that among all major clades of terrestrial plants, the upper limit of leaf surface conductance to CO_2_ and water vapor is tightly coupled to the biophysical limitations of cell size [8–11]. Cellular allometry, in particular the scaling of genome size, nuclear volume, and cell size, represents a direct physical constraint on the number of cells that can occupy a given space and, as a result, on the distance between cell types and tissues [12–14]. Because leaves with many small stomata and a high density of veins can maintain higher rates of gas exchange than leaves with fewer, larger stomata and larger, less numerous veins [15], variation in cell size can drive large changes in potential carbon gain. Without reducing cell size, increasing stomatal and vein densities would displace other important tissues, such as photosynthetic mesophyll cells [16]. Therefore, the densities of stomata on the leaf surface and of veins inside the leaf are inversely related to the sizes of guard cells and the primary xylem elements comprising them.

While numerous environmental and physiological factors can influence the final sizes of somatic eukaryotic cells, the minimum size of meristematic cells and the rate of their production are strongly constrained by nuclear volume, more commonly measured as genome size [17–19]. Among land plants, the bulk DNA content of cells varies by three orders of magnitude, with the angiosperms exhibiting both the largest range in genome size and the smallest absolute genome sizes [20]. Whole-genome duplications and subsequent genomic rearrangements, including genome downsizing, are thought to have directly contributed to the unparalleled diversity in anatomical, morphological, and physiological traits of the angiosperms [12,21–28]. We extend this prior work and test the hypothesis that genome size variation is responsible not only for gene diversification but also directly limits minimum cell size and, thus, is the underlying variable constraining stomatal size and density and leaf vein density (*D*_*v*_). Due to the strong influence of cell size on maximum potential carbon gain, the allometric scaling of genome size and cell size is predicted to directly influence primary productivity across all major clades of terrestrial plants [12,13,27,29].

## Results and discussion

**Fig 1:**
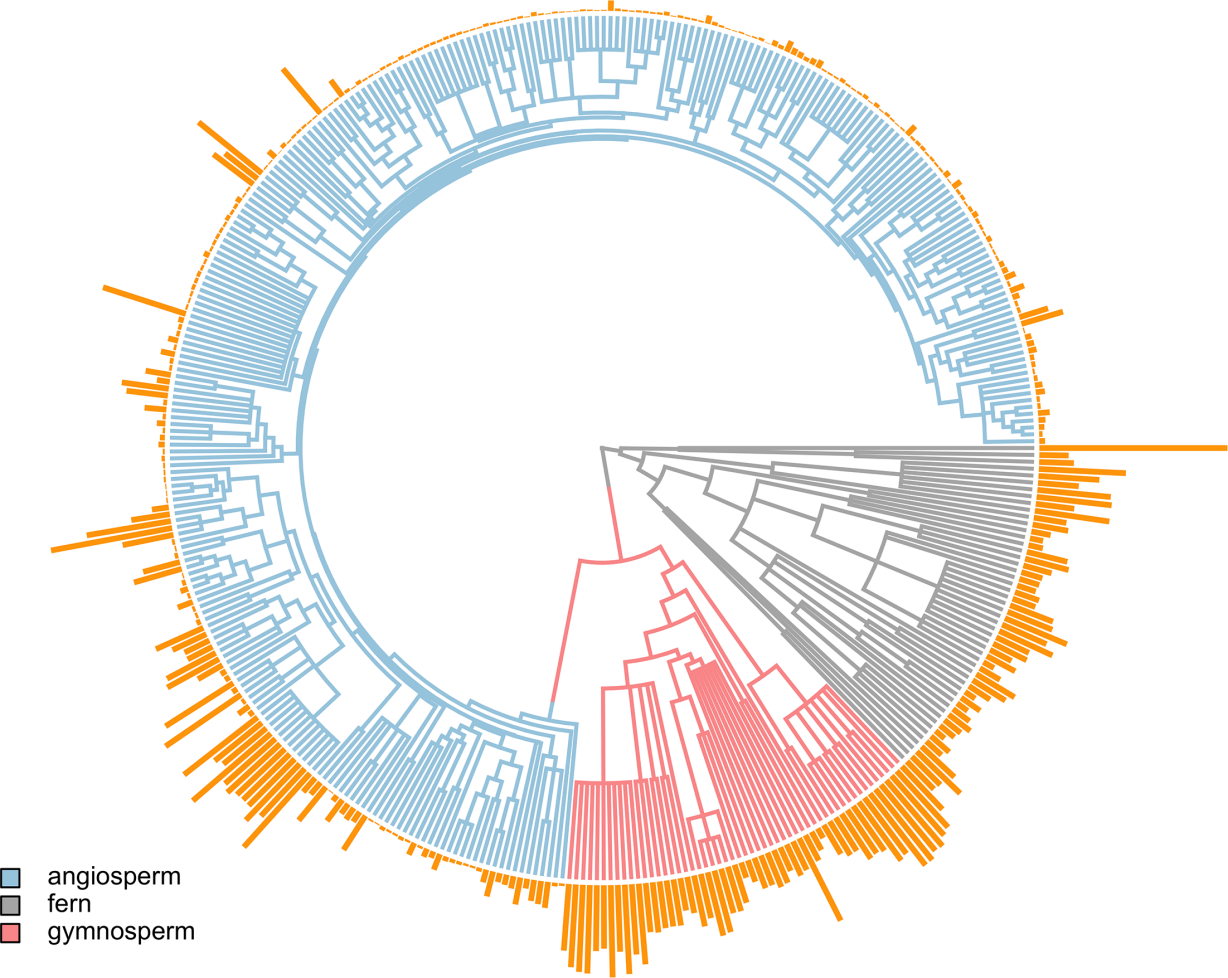
The distribution of genome size among 393 land plant species. Branch lengths are colored according to clade (ferns, gymnosperms, angiosperms). Orange bars at the tips are scaled proportional to genome size for each terminal species. Data can be found in S1 Data.

**Fig 2:**
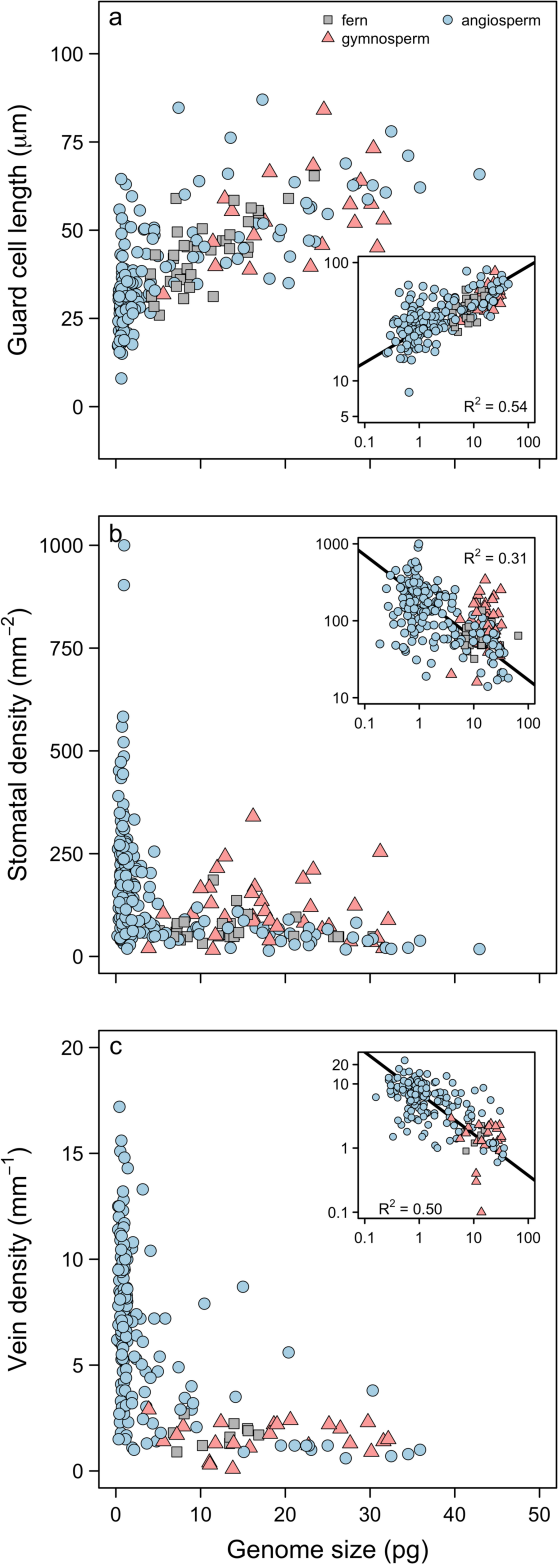
Relationships between genome size and anatomical traits: (a) *l*_*g*_, (b) *D*_*s*_, and (c) *D*_*v*_. In all panels, insets show log-log relationships and *R*^2^ values are from standard major axis regressions (*l*_g_ *n* = 242; *D*_s_ *n* = 247; *D*_v_ *n* = 198). Phylogenetically corrected major axis regressions have similar slopes, *R*^*2*^, and *p*-values, and are shown in Table 1. Data can be found in S1 Data. *D*_*s*_, stomatal density; *D*_*v*_, leaf vein density; *l*_*g*_, guard cell length.

To determine whether genome downsizing among the angiosperms drove the anatomical and physiological innovations that resulted in their ecological dominance, we compiled data for genome size, cell size (guard cell length; *l*_*g*_), stomatal density (*D*_*s*_), and *D*_*v*_ for almost 400 species of ferns, gymnosperms, and angiosperms. Consistent with prior studies and with our predictions, genome size varied substantially among major clades (Fig 1) and was a strong predictor of anatomical traits across the major groups of terrestrial plants even after accounting for phylogenetic relatedness of species (Fig 2, Table 1). Species with smaller genomes have smaller, more numerous stomata and higher leaf vein densities. Genome size explained between 31% and 54% of interspecific variation in *l*_g,_ *D*_*s*_, and *D*_*v*_ across the major groups of terrestrial plants, and both phylogenetic and non-phylogenetic analyses showed that a single relationship predicted each of these traits from genome size across all species (Table 1). In both phylogenetic and non-phylogenetic analyses there were strong, significant correlations between anatomical traits both among the major clades and within the angiosperms, highlighting the coordinated evolution of these traits throughout the history of seed plants (S1 Table).

**Table 1:** Non-phylogenetic standard major axis regressions of *D*_*v*_, *l*_*g*_, *D*_*s*_, *g*_s, max_, and *g*_s, op_ versus genome size for all species and for each clade separately and phylogenetic standard major axis regressions. Asterisks indicate significance level: *p < 0.05; ***p < 0.001. No regressions were significant with *p* < 0.01.

	all			angiosperm			gymnosperm			fern		
Non-phylogenetic												
	slope	elevation	R^2^	slope	elevation	R^2^	slope	elevation	R^2^	slope	elevation	R^2^
*D*_v_	−0.641 (−0.709, −0.580)	0.855 (0.808, 0.903)	0.497***	−0.628 (−0.710, −0.555)	0.847 (0.800, 0.893)	0.368***	1.34 (0.864, 2.08)	−1.488 (−2.244, −0.732)	0.002	0.937 (0.453, 1.94)	−0.775 (−1.58, 0.025)	0.054
*l*_g_	0.272 (0.250, 0.296)	1.43 (1.41, 1.45)	0.544***	0.294 (0.265, 0.326)	1.438 (1.417, 1.459)	0.497***	0.529 (0.354, 0.792)	1.04 (0.749, 1.32)	0.300*	0.489 (0.387, 0.617)	1.15 (1.04, 1.27)	0.514***
*D*_*s*_	−0.541 (−0.600, −0.488)	2.31 (2.26, 2.36)	0.314***	−0.615 (−0.690, −0.548)	2.26 (2.21, 2.31)	0.373***	1.53 (1.09, 2.15)	0.071 (−0.592, 0.733)	0.000	−0.674 (−1.02, −0.448)	2.56 (2.22, 2.89)	0.003
*g*_s, max_	−0.449 (−0.509, −0.395)	0.215 (0.168, 0.261)	0.240***	−0.438 (−0.505, −0.380)	0.200 (0.152, 0.247)	0.167***	1.18 (0.668, 2.08)	−1.75 (−2.65, −0.854)	0.004	0.971 (0.425, 2.22)	−1.20 (−2.11, −0.288)	0.352
*g*_s, op_ 70 μm	−0.687 (−0.761, −0.621)	−0.571 (−0.623, −0.520)	0.468***	−0.637 (−0.719, −0.564)	−0.581 (−0.627, −0.534)	0.381***	1.63 (1.05, 2.54)	−3.32 (−4.24, −2.40)	0.002	1.17 (0.565, 2.41)	−2.47 (−3.46, −1.48)	0.060
*Phylogenetic*												
*D*_v_	−0.766	0.958	0.122***									
*l*_g_	0.371	1.27	0.370***									
*D*_*s*_	−0.764	2.65	0.203***									
*g*_s,max_	−0.574	0.361	0.055***									
*g*_s,op,_ 70 μm	−0.779	−0.489	0.117***									

**Abbreviations:** *D*_*s*_, stomatal density; *D*_*v*_, leaf vein density; *g*_*s*, max_, maximum stomatal conductance; *g*_*s*, op_, operational stomatal conductance; *l*_*g*_, guard cell length.

Because genome size directly affects minimum cell size, variation in genome size has numerous consequences for the structure and organization of cells and tissues in leaves, which directly influence rates of leaf water loss (transpiration) and photosynthesis. Physical resistance to diffusion across leaf surfaces is ultimately determined by the sizes of epidermal cells, and the maximum diffusive conductance of CO_2_ and water vapor is higher in leaves with more numerous, smaller stomata [8,10,11]. While the effects of cell size on leaf epidermal properties have been well characterized, the effects of cell size on the efficiency of liquid water supply through the leaf are, perhaps, less obvious. Because the highest hydraulic resistance in the leaf occurs in the path between the veins and the sites of evaporation, shortening this path length by increasing *D*_*v*_ reduces the resistance outside the xylem and increases leaf hydraulic conductance [7,30]. Given a constant leaf volume, increasing *D*_v_ without displacing photosynthetic mesophyll cells requires reductions in vein and conduit sizes that can only be accomplished by decreasing cell size [16,31]. However, smaller conduits have higher hydraulic resistances. To overcome hydraulic limitations associated with reductions in conduit size, other innovations in xylem anatomy that reduce hydraulic resistance have been hypothesized to facilitate narrower xylem conduits and high *D*_v_. In particular, the development of low resistance end walls between adjacent cells is thought to have given angiosperms a hydraulic advantage as conduit diameters decreased. Only in angiosperm lineages with very high *D*_v_ do primary xylem have simple perforation plates, which have lower resistance to water flow than scalariform perforation plates [16]. Similarly, the low resistance of gymnosperm torus-margo pits compared to angiosperm pits can result in higher xylem-specific hydraulic conductivity for small diameter conduits [32]. In both cases, while smaller conduits have higher resistance, this potential cost has been offset by other innovations that reduce hydraulic resistance at the scale of the whole xylem network.

**Fig 3:**
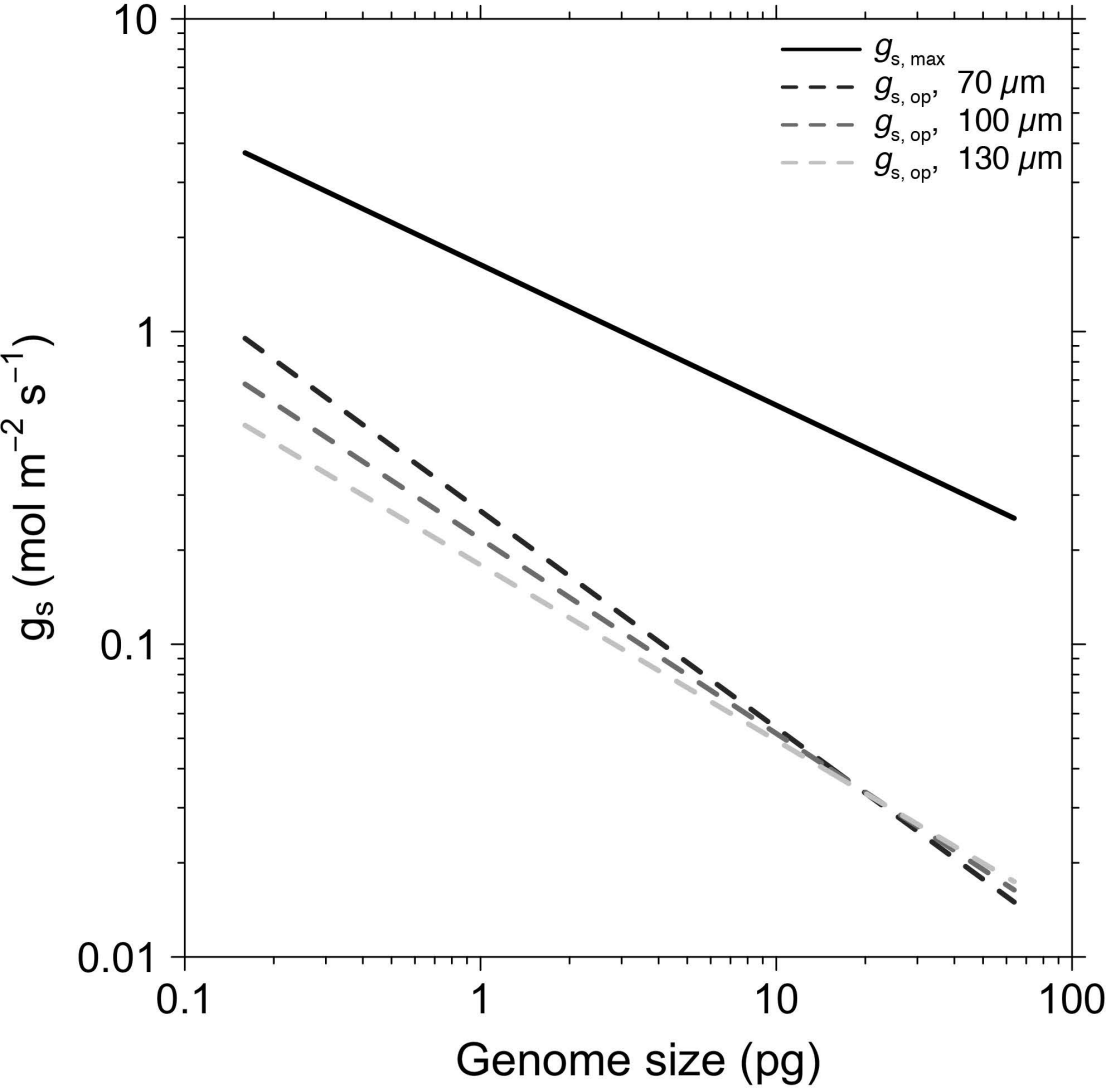
The major axis regressions between genome size and *g*_s_, _max_ (solid line; *R*^2^ = 0.24, *n* = 184) and *g*_s, op_, operational stomatal conductance (dashed lines; *n* = 198), plotted on a log-log scale. *g*_s, op_ was calculated using a hydraulic model based on vein spacing under assumptions of three leaf thicknesses (70 μm, *R*^2^ = 0.47; 100 μm, *R*^2^ = 0.45; 130 μm, *R*^2^ = 0.44; see Eqs 2–6 for details) and an assumed vapor pressure deficit of 2 kPa. Variation in vapor pressure deficit will affect the intercept of *g*_s, op_ but not the slope. Points are omitted for clarity. Phylogenetically corrected major axis regressions are similarly significant and are reported in Table 1. *g*_s_, _max,_ maximum stomatal conductance; *g*_s, op_, operational stomatal conductance.

We examined the consequences of variation in genome size on terrestrial primary productivity by calculating maximum stomatal conductance (*g*_s, max_) and operational stomatal conductance (*g*_s, op_) using theoretical and empirical models that directly relate leaf anatomy to gas exchange (see Materials and Methods). Genome size was a highly significant predictor of both *g*_s, max_ and *g*_s, op_, whether or not phylogenetic relatedness of species was incorporated (Fig 3, Table 1). Scaling relationships that accounted for phylogenetic relatedness of all species in our dataset were as significant as non-phylogenetic analyses and had similar slopes. Thus, a single relationship between genome size and stomatal conductance exists among all land plants. We tested assumptions about how vein positioning in the leaf influences *g*_s, op_ by modeling stomatal conductance for leaves of varying thickness and found that regardless of leaf thickness (70, 100, 130 μm), the slopes of the relationships between genome size and *g*_s, op_ were significantly steeper than the slope of the relationship between genome size and *g*_s, max_ (all *p* < 0.001). Thus, across all species, shrinking the genome brings *g*_s, op_ closer to *g*_s, max_ (Fig 3, Table 1), which facilitates faster rates of growth.

**Fig 4:**
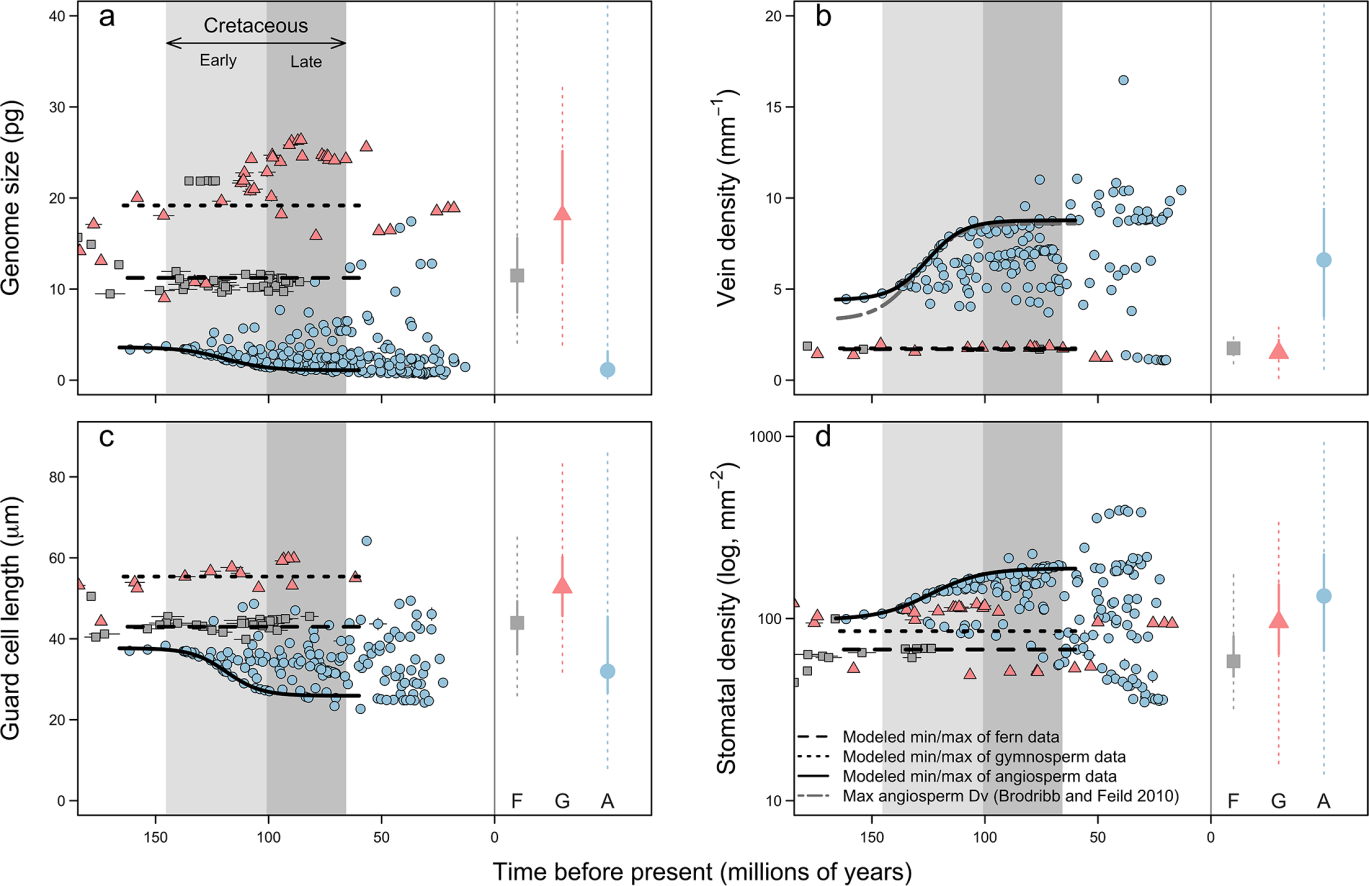
Ancestral state reconstructions of genome size, *D*_*v*_, *l*_*g*_, and *D*_*s*_ through time for angiosperms (A; blue circles), gymnosperms (G; pink triangles), and ferns (F; grey squares). Error bars around reconstructed values represent error due to phylogenetic uncertainty. The shaded timespan indicates the Cretaceous period, during which most major lineages of angiosperms diversified. Lines represent the best-fit models through the lower (genome size and *l*_*g*_) and upper (*D*_*v*_ and *D*_*s*_) 10% of reconstructed values. For all traits, extreme trait values of angiosperms rapidly changed during the Cretaceous period, whereas fern and gymnosperm lineages underwent no such changes. (a) Genome size of ferns (genome size = 10.24; *n* = 51, df = 11, *p* < 0.001), gymnosperms (genome size = 19.46; *n* = 53, df = 13, *p* < 0.001), and angiosperms (genome size = 1.08 + 2.53/(1 + e^(−(time − 119.28) / 9.52; *n* = 289, df = 20, *p* < 0.001). (b) Leaf *D*_*v*_ of ferns (*D*_*v*_ = 1.70; *n* = 10, df = 1, *p* < 0.01), gymnosperms (*D*_*v*_ = 1.74; *n* = 23, df = 9, *p* < 0.001), and angiosperms (*D*_*v*_ = 4.39 + 4.42 / (1 + e^(−[time − 125.32] / [−8.22]), *n* = 165, df = 17, *p* < 0.001). (c) *l*_*g*_ of ferns (*l*_*g*_ = 43.04; *n* = 38, df = 12, *p* < 0.001), gymnosperms (*l*_*g*_ = 55.48; *n* = 20, df = 8, *p* < 0.001), and angiosperms (*l*_*g*_ = 26.93 + 10.58 / (1 + e^(−[time − 119.73] / 6.12); *n* = 184, df = 16, *p* < 0.001. (d) *D*_*s*_ of ferns (*D*_*s*_ = 1.80; *n* = 26, df = 2, *p* < 0.001), gymnosperms (*D*_*s*_ = 1.94; *n* = 37, df = 11, *p* < 0.001), and angiosperms (*D*_*s*_ = 2.33 − 0.364 / (1 + e^(−[time − 121.65] / 17.39); *n* = 184, df = 15, *p* < 0.001). Marginal plots on the outside of each panel represent the median (points), interquartile ranges (solid lines), and ranges (dotted lines) of trait values for extant species. A, angiosperms; *D*_*s*,_ stomatal density; *D*_*v*_, vein density; F, ferns; G, gymnosperms; *l*_*g*,_ guard cell length.

The timing of these physiological innovations further corroborates their role in promoting angiosperm domination of terrestrial ecosystems. Unlike other major clades of terrestrial plants, genome sizes, *D*_v_, *D*_*s*_, and *l*_g_ of the angiosperms expanded into new regions of trait space during the Cretaceous period (Fig 4), increasing rates of leaf level carbon assimilation and ushering in an era of greater terrestrial primary productivity [12,15,27]. To determine how the upper or lower limits of trait values changed through time, linear and nonlinear curves were fit through the upper or lower 10% of trait values during the period of rapid angiosperm diversification (165–60 Ma). For the angiosperms, extreme values of genome size and anatomical traits were fit by a logarithmic curve better than by a linear relationship (genome size change in Akaike Information Criterion (**Δ**AIC) = 31.8; *D*_v_ **Δ**AIC = 6.6; *l*_g_ **Δ**AIC = 16.3; *D*_*s*_ **Δ**AIC = 5.7), indicating that Cretaceous angiosperms pushed the frontiers of genome size, cell size, and vein and stomatal densities. In contrast to the angiosperms, fern and gymnosperm lineages exhibited no such sudden change in any trait during the Cretaceous period (Fig 4). Reconstructions of *D*_*v*_ matched well with fossil data, but the limited available data for *l*_*g*_ and *D*_*s*_ among Cretaceous angiosperms precludes a comparable analysis (S1 Fig).

These results suggest that the ability to develop leaves with high vein and stomatal densities derives not exclusively from common developmental programs underlying these traits nor from genetic correlations (i.e., linkage between genes controlling both traits), but—even more fundamentally—from biophysical scaling constraints that limit minimum cell size [8,29]. Together with analyses of trait evolution, the scaling relationships between genome size and gas exchange rates suggest that rapid genome downsizing among the angiosperms during the Cretaceous period facilitated increased rates of photosynthesis and biomass accumulation (Fig 2, Fig 3 and Fig 4). Importantly, while genome downsizing has been critical to increasing leaf gas exchange rates among the angiosperms, it was not a key innovation that occurred only at the root of the angiosperm phylogeny. Rather, the angiosperms exhibit a wide range of genome sizes, and coordinated changes in genome size and physiological traits have occurred repeatedly throughout their evolutionary history (Table 1, S1 Table). While whole-genome duplications have been particularly important in promoting diversification among the angiosperms [21], larger genomes increase minimum cell size and depress maximum potential gas exchange, thereby reducing competitive ability in productive habitats. Our results suggest that reductions in minimum cell size through genome downsizing can recover leaf gas exchange capacity subsequent to genome duplications and diversification events. If heightened competitive ability among the angiosperms drove their ecological dominance, then innovations that reduced minimum cell size were critical to this transformative process [29].

Although genome size limits minimum cell size [19,25], final cell size can vary widely as cells grow and differentiate. After cell division and during cell expansion, various factors influence how large a cell becomes. Intracellular turgor pressure overcomes the mechanical rigidity of the cell wall to enlarge cellular boundaries. The magnitude of turgor pressure is itself controlled by water availability around the cell and by the osmotic potential inside the cell. Final cell size is influenced, therefore, by both biotic and abiotic factors that affect pressure gradients in and around the cell. By reducing nuclear volume and the lower limit of cell size [19,25], genome downsizing expands the range of final cell size that is possible. Species that can vary cell size across a wider range can more finely tune their leaf anatomy to match environmental constraints on leaf gas exchange. Indeed, *D*_v_, *D*_*s*_, *l*_g_, and *g*_*s*_ are more variable among species with small genomes (Fig 2, Fig 3 and Fig 4), and the variance in these traits unexplained by genome size is likely due to environmental variation. Thus, genome size may predict ecological breadth insofar as species with small genomes can exhibit greater plasticity in final cell size and can inhabit a wider range of environmental conditions, although more analyses of within- and between-species variation in genome size are needed to clarify this [33,34]. Interestingly, only the angiosperms occupy this region of trait space, and the angiosperms tend to be more productive than either the ferns or the gymnosperms across a broad range of environmental conditions. Therefore, rapid genome downsizing by the angiosperms during the Cretaceous period likely explains not only their greater potential and realized primary productivity (Fig 3 and Fig 4) but also why they were able to expand into and create new ecological habitats, fundamentally altering the global biosphere and atmosphere [3].

Prevailing theories have suggested that the global dominance of angiosperms occurred due to higher maximum photosynthetic capacity and growth, despite Cretaceous declines of atmospheric CO_2_ that would have otherwise depressed rates of photosynthesis [3,12,15,35]. In habitats that can support high rates of primary productivity, maximum rates of gas exchange and growth are generally greater for angiosperms than for gymnosperms and ferns and are due, we show, to reductions in genome and cell sizes that occurred after the appearance of early angiosperms. Smaller genomes and cells increased leaf surface conductance to CO_2_ and enabled higher potential and realized primary productivity. Furthermore, because genome downsizing lowers the limit of minimum cell size, final cell size can vary much more widely, which may facilitate a closer coupling of anatomy and physiology to environmental conditions [36]. Therefore, genome downsizing among the angiosperms allowed them to outcompete other plants in almost every terrestrial ecosystem.

## Materials and methods

### Leaf traits

Published data for *l*_g_, *D*_s_, and *D*_v_ were compiled from the literature (S1 Data). Genome size data for each species were taken from the Plant DNA C-values database (release 6.0, December 2012), managed by the Royal Botanic Gardens, Kew [37]. In total, our dataset comprised 393 species of vascular plants, of which 289 were angiosperms, 53 were gymnosperms, and 51 were ferns. The dataset comprised here represents 0.1% of the estimated angiosperm species diversity. Of the 416 families and 64 orders of extant plants currently accepted by the Angiosperm Phylogeny Group IV, the 289 species in our dataset represented 102 families and 43 orders. Among angiosperm clades, the species diversity in our dataset is positively correlated with the number of known species in those clades (S2 Fig). The Plant DNA C-values database currently contains data for over 7,000 angiosperms, and our sample of 289 for which there were anatomical traits had genome sizes highly representative of all angiosperms in the database with no significant differences between the mean genome sizes of the two datasets (S3 Fig). For the 289 angiosperms in the dataset, there were *D*_v_ data for 165, guard cell size data for 184, and *D*_*s*_ data for 184. Similarly, there were *D*_v_ data for 23 gymnosperms and for 10 ferns, there were *l*_g_ data for 20 gymnosperms and for 38 ferns, and there were *D*_*s*_ data for 37 gymnosperms and 26 ferns.

Fossil data for *D*_*v*_ [38,39], *l*_*g*_ [8,27,40,41], and *D*_*s*_ [8,40] were compiled from published sources (S1 Data).

### Calculating *g*_s, max_ and *g*_s, op_

For each species, we calculated *g*_s, max_ and *g*_s, op_. *g*_s, max_ is defined by the dimensions of stomatal pores and their abundance, and represents the biophysical upper limit of gas diffusion through the leaf epidermis. Anatomical measurements of guard cells were used to calculate *g*_s, max_ as [8,9]:

$$g_{s,\;\max}=\frac{D_{s}a_{\max}\frac{d_{H_{2}O}}{m_{v}}}{d_{p}+\frac{\pi }{2}\sqrt{a_{\max}/\pi }}$$ (1)

where $d_{H_{2}O}$ is the diffusivity of water in air (0.0000249 m^2^s^−1^), *m*_v_ is the molar volume of air normalized to 25°C (0.0224 m^3^mol^−1^), *D*_s_ is stomatal density (mm^−2^), *a*_max_ is maximum stomatal pore size, and *d*_p_ is the depth of the stomatal pore. The *a*_max_ term can be approximated as: π(*l*_p_/2)^2^, where *l*_p_ is stomatal pore length with *l*_p_ being approximated as *l*_g_/2, where *l*_g_ is guard cell length. For studies that only reported *l*_p_, we calculated *l*_g_ as 2∙*l*_p_ [8,42]. *d*_p_ is assumed to be equal to guard cell width (*W*). If *W* was not reported *d*_p_ was estimated as 0.36∙*l*_*g*_ [11].

*g*_s, op_, by contrast, more accurately defines the stomatal conductance leaves attained under natural conditions when limitations in leaf hydraulic supply constrain stomatal conductance. We used an empirical model of *g*_*s*, op_ that directly relates *D*_v_ to stomatal conductance during periods of steady state transpiration (*E*) [7] as:

$$E=g_{s,\;op}v=K_{\mathrm{leaf}}\Delta \Psi$$ (2)

$$K_{\mathrm{leaf}}=12,670d_{m}{}^{\text{-}1.27}$$ (3)

where:

$$d_{m}=\pi /2\;{\left(d_{x}{}^{2}+d_{y}{}^{2}\right)}^{1/2}$$ (4)

$$d_{x}=650/D_{v}$$ (5)

$$g_{s,\;op}=(K_{\mathrm{leaf}}\;\Delta \Psi )/v.$$ (6)

*K*_leaf_ is leaf hydraulic conductance (mmol m^−2^s^−1^MPa^−1^), *d*_m_ is the post vein distance to stomata (μm), *d*_x_ is the maximum horizontal distance from vein to the stomata (μm), *d*_y_ is the distance from vein to the epidermis (μm), ΔΨ is the water potential difference between stem and leaf (set to 0.33 MPa [43]), and *v* is vapor pressure deficit set to 2 kPa. Variation in *v* would affect the intercept but not the slope of *g*_s, op_. In order to test the influence of variation in leaf thickness on *g*_s, op_, we used three values of *d*_y_ (70, 100, and 130 μm). The steady state equations presented above can be related directly to photosynthesis as:

Aop=E1.6v(ca(1−cica))=(KLeafΔΨ)1.6v(ca(1−cica))=gs,op1.6(ca(1−cica)) (7)

where *A*_op_ is operational photosynthetic capacity (μmol m^−2^s^−1^), *c*_a_ is the molar concentration of CO_2_ in the atmosphere, *c*_i_ is the molar concentration of CO_2_ in the air spaces inside the leaf, and 1.6 accounts for the difference in diffusivity between H_2_O and CO_2_ in air.

### Analyses of trait evolution

To determine the temporal patterns of trait evolution, we generated a phylogeny from the list of taxa (S1 Data) using Phylomatic (v. 3) and its stored family-level supertree (v. R20120829). To date nodes in the supertree, we compiled node ages from recent, fossil-calibrated estimates of crown group ages. Node ages were taken from Magallón et al. [44] for angiosperms, Lu et al. [45] for gymnosperms, and Testo and Sundue [46] for ferns. The age of all seed plants was taken as 330 million years [47]. Because there is some uncertainty in the maximum age of the ancestor of all angiosperms, we took the angiosperm crown age used by Brodribb and Field [12] to make our results directly comparable to theirs. We tested this assumed angiosperm age by using different ages for the crown group angiosperms ranging from 130 Ma to 180 Ma, and the results were not qualitatively different. Of the 254 internal nodes in our tree, 82 of them had ages. These ages were assigned to nodes and branch lengths between these dated nodes evenly spaced using the function “bladj” in the software Phylocom (v. 4.2 [47]). Polytomies were resolved by randomly bifurcating and adding 5 million years to each of these new branches and subtracting an equivalent amount from the descending branches so that the tree remained ultrametric. For all subsequent analyses of character evolution, this method for randomly resolving polytomies was repeated 100 times to account for phylogenetic uncertainty. For ancestral state reconstructions, the ages and character estimates at each node were averaged across the 100 randomly resolved trees.

Ancestral state reconstructions were calculated using the residual maximum likelihood method, implemented in the function “ace” from the R package *ape* [48]. To determine when changes in traits pushed the frontiers of trait values, the upper (*D*_v_ and *D*_*s*_) and lower (genome size and *l*_g_) limits of traits were estimated by first extracting the upper or lower ten percent of reconstructed trait values in sequential 5 million-year windows and then attempting to fit curves to these values. This method is similar to a previous analysis of *D*_v_ evolution through time [38], which is included here for comparison. We compared three types of curve fits: a linear fit that lacked slope (equivalent to the mean of the reconstructed trait values), a linear fit that included both a slope and an intercept, and a nonlinear curve of the form *trait* = *a* + *b* / (1 + *e*^[−(*time* + *c*) / *d*]). Curves were fit to reconstructed trait values for each clade between 165 and 60 Ma, which corresponds to the time period encompassing the major diversification and expansion of the angiosperms, and the best fit was chosen based on AIC scores with a difference in AIC of 5 taken to indicate significant differences in fits. Phylogenetic generalized least squares regression was used to determine whether traits underwent correlated evolution. A regression was performed for each pairwise combination of traits for only species with data for both traits. Phylogenetic regressions used a Brownian motion correlation structure from the R package *ape* [49].

We acknowledge the potential for high uncertainty in ancestral state character reconstructions when working with a small subset of species relative to the broader species pool [50,51]. In an effort to minimize uncertainty, we sampled basal angiosperms as much as possible and performed two additional analyses that suggest our dataset is robust to incomplete sampling. First, we performed a bootstrapping analysis in which we randomly sampled species from our entire genome size dataset (35%, 52%, and 78% of angiosperm species), reconstructed genome size, and fit curves to the lower limit of reconstructed genome sizes, as before. This procedure was replicated 100 times at each level of sampling diversity. This analysis revealed that using only 35% of the angiosperms in our dataset still produced estimates of minimum genome size that are consistent with the entire dataset (S4 Fig). Second, the species diversity of 20 named nodes in our dataset is strongly correlated with the actual extant species diversity of those clades (S2 Fig). Additionally, our sample of genome size variation does not differ significantly from the genome size variation among approximately 7,000 measured species (S3 Fig). Furthermore, our analysis of vein density evolution based on 151 angiosperm species is almost identical to the previous analysis by Brodribb and Feild [12], which relied on 504 angiosperm species (Fig 4), and both of these modeled limits of vein density agree strongly with fossil data [38]. Overall, these analyses strongly suggest that the trait values represented in our taxon sampling is robust, given the incredible extant diversity of angiosperms and the data currently available.

### Scaling relationships

Scaling relationships between genome size and *D*_v_, *l*_g_, *g*_s, max_, and *g*_s, op_ were calculated from log-transformed data and analyzed using the function “sma” in the R package *smatr* [52]. Analyses were performed for the entire dataset and also for individual clades. Slope tests were used to determine whether the scaling relationship between genome size and *g*_s, max_ was significantly different from the relationship between genome size and *g*_s, op_ and whether the scaling relationships between genome size and *g*_s, op_ and *g*_s, max_ differed among clades. To account for the non-independence of sampling related species, phylogenetic standard major axis regressions were performed on all species using the function “phyl.RMA” in the R package *phytools*.

## Supporting information

S1 Table *l*_*g*_, *D*_*s*_, and *D*_*v*_ for species used in the analysis. *D*_*s*_, stomatal density; *D*_*v*_, vein density; *l*_*g*_, guard cell length.
(DOCX)

S2 Table Trait and PGLS regressions for all species and for only the angiosperms. Trait regressions are in the upper triangle and PGLS regressions are in the lower triangle. Values are regression slopes. Asterisks indicate significance level: *p < 0.05; **p < 0.01; ***p < 0.001. PGLS, phylogenetic generalized least squares.
(DOCX)

S1 Data *l*_*g*_, *D*_*s*_, and *D*_*v*_ for species used in the analysis. *D*_*s*_, stomatal density; *D*_*v*_, vein density; *l*_*g*_, guard cell length.
(XLSX)

S1 Fig Fossil data of anatomical traits plotted with limits of trait values reconstructed from extant species (curves from Fig 4). Data can be found in S1 Data.
(TIF)

S2 Fig The number of currently accepted species for 20 named clades in our phylogeny is strongly correlated with the number of species representing those clades in our dataset (*r* = 0.69, *p* < 0.001). (TIFF)

S3 Fig The distributions of genome size among angiosperms in the Kew plant DNA C-values database, which includes over 7,000 species, and of species sampled in the present study are not significantly different (*t* = 1.69, *p* = 0.1). (a) Untransformed distributions and (b) Log-transformed distributions. In both figures, the number of species for the Kew database is on the left axis, and the number of species sampled in this study is on the right axis.
(TIFF)

S4 Fig Bootstrapping analyses of the lower limit of genome size modeled from ancestral state reconstructions based on 35%, 52%, and 78% of angiosperm species in our entire dataset. Heavy black lines are the modeled limit from the entire dataset, and the light grey, red, and blue lines are the modeled limits from each of 100 replicate runs at each level of diversity. The modeled limits of genome size for ferns (dashed lines) and gymnosperms (dotted lines) are reported from Fig 4A.
(TIFF)

## Abbreviations

AIC: Akaike Information Criterion
*c*_i_: leaf intercellular CO_2_ concentrations
*D_s_*: stomatal density
*D_v_*: leaf vein density
*g*_s, max_: maximum stomatal conductance
*g*_s, op_: operational stomatal conductance
l_g_: guard cell length
W: guard cell width

## References

[1] Lee J-E, Boyce K. Impact of the hydraulic capacity of plants on water and carbon fluxes in tropical South America. Journal of Geophysical Research. 2010;115: D23123 doi: 10.1029/2010JD014568

[2] Boyce CK, Lee J-E. Could land plant evolution have fed the marine Revolution? Paleontological Research. 2011;15: 100–105. doi: 10.2517/1342-8144-15.2.100

[3] Boyce CK, Brodribb TJ, Feild TS, Zwieniecki MA. Angiosperm leaf vein evolution was physiologically and environmentally transformative. Proceedings of the Royal Society B. 2009;276: 1771–1776. doi: 10.1098/rspb.2008.1919 19324775 10.1098/rspb.2008.1919 PMC2674498

[4] Ehleringer JR, Monson RK. Evolutionary and ecological aspects of photosynthetic pathway variation. Annual Review of Ecology and Systematics 1993;24: 411–439.

[5] Cowan IR. Stomatal behaviour and environment. Advances in Botanical Research. Elsevier; 1977;4: 117–228. doi: 10.1016/S0065-2296(08)60370-5

[6] Wong S, Cowan I, Farquhar G. Stomatal conductance correlates with photosynthetic capacity. Nature. 1979;282: 424–426.

[7] Brodribb TJ, Feild TS, Jordan GJ. Leaf maximum photosynthetic rate and venation are linked by hydraulics. Plant Physiology. 2007;144: 1890–1898. doi: 10.1104/pp.107.101352 17556506 10.1104/pp.107.101352 PMC1949879

[8] Franks PJ, Beerling DJ. Maximum leaf conductance driven by CO_2_ effects on stomatal size and density over geologic time. Proceedings of the National Academy of Sciences. 2009;106: 10343–10347. doi: 10.1073/pnas.0904209106 19506250 10.1073/pnas.0904209106 PMC2693183

[9] Franks PJ, Farquhar GD. The effect of exogenous abscisic acid on stomatal development, stomatal mechanics, and leaf gas exchange in *Tradescantia virginiana*. Plant Physiology. 2001;125: 935–942. 11161050 10.1104/pp.125.2.935 PMC64894

[10] Sack L, Buckley TN. The developmental basis of stomatal density and flux. Plant Physiology. 2016;171: 2358–2363. doi: 10.1104/pp.16.00476 27268500 10.1104/pp.16.00476 PMC4972277

[11] de Boer HJ, Price CA, Wagner-Cremer F, Dekker SC, Franks PJ, Veneklaas EJ. Optimal allocation of leaf epidermal area for gas exchange. New Phytologist. 2016;210: 1219–1228. doi: 10.1111/nph.13929 26991124 10.1111/nph.13929 PMC5069575

[12] Brodribb TJ, Feild TS. Leaf hydraulic evolution led a surge in leaf photosynthetic capacity during early angiosperm diversification. Ecology Letters. 2010;13: 175–183. doi: 10.1111/j.1461-0248.2009.01410.x 19968696 10.1111/j.1461-0248.2009.01410.x

[13] Brodribb TJ, Jordan GJ, Carpenter RJ. Unified changes in cell size permit coordinated leaf evolution. New Phytologist. 2013;199: 559–570. doi: 10.1111/nph.12300 23647069 10.1111/nph.12300

[14] John GP, Scoffoni C, Sack L. Allometry of cells and tissues within leaves. American Journal of Botany. 2013;100: 1936–1948. doi: 10.3732/ajb.1200608 24070860 10.3732/ajb.1200608

[15] de Boer HJ, Eppinga MB, Wassen MJ, Dekker SC. A critical transition in leaf evolution facilitated the Cretaceous angiosperm revolution. Nature Communications. Nature Publishing Group; 2012;3: 1–11. doi: 10.1038/ncomms2217 23187621 10.1038/ncomms2217 PMC3514505

[16] Feild TS, Brodribb TJ. Hydraulic tuning of vein cell microstructure in the evolution of angiosperm venation networks. New Phytologist. 2013;199: 720–726. doi: 10.1111/nph.12311 23668223 10.1111/nph.12311

[17] Mirsky AE, Ris H. The deoxyribonucleic acid content of animal cells and its evolutionary significance. The Journal of General Physiology. 1951;34: 451–462. 14824511 10.1085/jgp.34.4.451 PMC2147229

[18] Cavalier-Smith T. Nuclear volume control by nucleoskeletal DNA, selection for cell volume and cell growth rate, and the solution of the DNA C-value paradox. Journal of Cell Science. 1978;34: 247–278. 372199 10.1242/jcs.34.1.247

[19] Simova I, Herben T. Geometrical constraints in the scaling relationships between genome size, cell size and cell cycle length in herbaceous plants. Proceedings of the Royal Society B. 2012;279: 867–875. doi: 10.1098/rspb.2011.1284 21881135 10.1098/rspb.2011.1284 PMC3259922

[20] Leitch IJ. Evolution of DNA amounts across land plants (Embryophyta). Annals of Botany. 2005;95: 207–217. doi: 10.1093/aob/mci014 15596468 10.1093/aob/mci014 PMC4246719

[21] Jiao Y, Wickett NJ, Ayyampalayam S, Chanderbali AS, Landherr L, Ralph PE, et al Ancestral polyploidy in seed plants and angiosperms. Nature;473: 97–100. doi: 10.1038/nature09916 21478875 10.1038/nature09916

[22] Lomax BH, Woodward FI, Leitch IJ, Knight CA, Lake JA. Genome size as a predictor of guard cell length in *Arabidopsis thaliana* is independent of environmental conditions. New Phytologist. 2009;181: 311–314. doi: 10.1111/j.1469-8137.2008.02700.x 19054335 10.1111/j.1469-8137.2008.02700.x

[23] Bennett MD, Leitch IJ. Nuclear DNA amounts in angiosperms: targets, trends and tomorrow. Annals of Botany. 2011;107: 467–590. doi: 10.1093/aob/mcq258 21257716 10.1093/aob/mcq258 PMC3043933

[24] Beaulieu JM, Leitch IJ, Patel S, Pendharkar A, Knight CA. Genome size is a strong predictor of cell size and stomatal density in angiosperms. New Phytologist. 2008;179: 975–986. doi: 10.1111/j.1469-8137.2008.02528.x 18564303 10.1111/j.1469-8137.2008.02528.x

[25] Bennett MD. Variation in genomic form in plants and its ecological implications. New Phytologist. 1987;106: 177–200.

[26] Hodgson JG, Sharafi M, Jalili A, Diaz S, Montserrat-Marti G, Palmer C, et al Stomatal vs. genome size in angiosperms: the somatic tail wagging the genomic dog? Annals of Botany. 2010;105: 573–584. doi: 10.1093/aob/mcq011 20375204 10.1093/aob/mcq011 PMC2850795

[27] Franks PJ, Freckleton RP, Beaulieu JM, Leitch IJ, Beerling DJ. Megacycles of atmospheric carbon dioxide concentration correlate with fossil plant genome size. Philosophical Transactions of the Royal Society B. 2012;367: 556–564. doi: 10.1038/338247a0 10.1098/rstb.2011.0269 PMC3248711 22232767

[28] Leitch IJ, Bennett MD. Genome downsizing in polyploid plants. Biological Journal of the Linnean Society. 2004;82: 651–663.

[29] Franks PJ, Leitch IJ, Ruszala EM, Hetherington AM, Beerling DJ. Physiological framework for adaptation of stomata to CO_2_ from glacial to future concentrations. Philosophical Transactions of the Royal Society B. 2012;367: 537–546. doi: 10.1073/pnas.0605618103 10.1098/rstb.2011.0270 PMC3248712 22232765

[30] Buckley TN, John GP, Scoffoni C, Sack L. How Does Leaf Anatomy Influence Water Transport outside the Xylem? Plant Physiology. 2015;168: 1616–1635. doi: 10.1104/pp.15.00731 26084922 10.1104/pp.15.00731 PMC4528767

[31] Coomes DA, Heathcote S, Godfrey ER, Shepherd JJ, Sack L. Scaling of xylem vessels and veins within the leaves of oak species. Biology Letters. 2008;4: 302–306. doi: 10.1098/rsbl.2008.0094 18407890 10.1098/rsbl.2008.0094 PMC2610058

[32] Pittermann J, Sperry J, Hacke U, Wheeler J, Sikkema E. Torus-Margo Pits Help Conifers Compete with Angiosperms. Science. 2005;310: 1924–1924. doi: 10.1126/science.1120479 16373568 10.1126/science.1120479

[33] Knight CA, Ackerly DD. Variation in nuclear DNA content across environmental gradients: a quantile regression analysis. Ecology Letters. 2002;5: 66–76.

[34] Hao G-Y, Lucero ME, Sanderson SC, Zacharias EH, Holbrook NM. Polyploidy enhances the occupation of heterogeneous environments through hydraulic related trade-offs in *Atriplex canescens* (Chenopodiaceae). New Phytologist. 2013;197: 970–978. doi: 10.1111/nph.12051 23206198 10.1111/nph.12051

[35] Bond WJ. The tortoise and the hare: ecology of angiosperm dominance and gymnosperm persistence. Biological Journal of the Linnean Society. 1989;36: 227–249.

[36] Carins Murphy MR, Jordan GJ, Brodribb TJ. Cell expansion not cell differentiation predominantly co-ordinates veins and stomata within and among herbs and woody angiosperms grown under sun and shade. Annals of Botany. 2016;118: 1127–1138. doi: 10.1093/aob/mcw167 27578763 10.1093/aob/mcw167 PMC5963197

[37] Bennett MD, Leitch IJ, editors. Plant DNA C-values database. 6 ed. kew; 2012 Available from: http://data.kew.org/cvalues/ Accessed 29 Sept 2016

[38] Feild T, Brodribb T, Iglesias A. Fossil evidence for Cretaceous escalation in angiosperm leaf vein evolution. Proceedings of the National Academy of Sciences, USA 2011;108: 8363–8366. 10.1073/pnas.1014456108 PMC3100944 21536892

[39] Boyce CK, Zwieniecki MA. Leaf fossil record suggests limited influence of atmospheric CO2 on terrestrial productivity prior to angiosperm evolution. Proceedings of the National Academy of Sciences, USA 2012;109: 10403–10408. doi: 10.1073/pnas.1203769109/-/DCSupplemental 10.1073/pnas.1203769109 PMC3387114 22689947

[40] Beerling D, Woodward F. Changes in land plant function over the Phanerozoic: Reconstructions based on the fossil record. Botanical Journal of the Linnean Society. 1997;124: 137–153.

[41] Lomax BH, Hilton J, Bateman RM, Upchurch GR, Lake JA, Leitch IJ, et al Reconstructing relative genome size of vascular plants through geological time. New Phytologist. 2013;201: 636–644. doi: 10.1111/nph.12523 24117890 10.1111/nph.12523

[42] Franks PJ, Farquhar GD. The mechanical diversity of stomata and its significance in gas-exchange control. Plant Physiology. 2007;143: 78–87. doi: 10.1104/pp.106.089367 17114276 10.1104/pp.106.089367 PMC1761988

[43] Simonin KA, Limm EB, Dawson TE. Hydraulic conductance of leaves correlates with leaf lifespan: implications for lifetime carbon gain. New Phytologist. 2012;193: 939–947. doi: 10.1111/j.1469-8137.2011.04014.x 22224403 10.1111/j.1469-8137.2011.04014.x

[44] Magallón S, Gómez-Acevedo S, Sánchez-Reyes LL, Hernández-Hernández T. A metacalibrated time-tree documents the early rise of flowering plant phylogenetic diversity. New Phytologist. 2015;207: 437–453. doi: 10.1111/nph.13264 25615647 10.1111/nph.13264

[45] Lu Y, Ran J-H, Guo D-M, Yang Z-Y, Wang X-Q. Phylogeny and Divergence Times of Gymnosperms Inferred from Single-Copy Nuclear Genes. Buerki S, editor. PLoS ONE. 2014;9: e107679 doi: 10.1371/journal.pone.0107679 25222863 10.1371/journal.pone.0107679 PMC4164646

[46] Testo W, Sundue M. Molecular Phylogenetics and Evolution. Molecular Phylogenetics and Evolution. Elsevier Inc; 2016;105: 200–211. doi: 10.1016/j.ympev.2016.09.003 27621129 10.1016/j.ympev.2016.09.003

[47] Webb CO, Ackerly DD, Kembel SW. Phylocom: software for the analysis of phylogenetic community structure and trait evolution. Bioinformatics. 2008;24: 2098–2100. doi: 10.1093/bioinformatics/btn358 18678590 10.1093/bioinformatics/btn358

[48] Paradis E, Claude J, Strimmer K. APE: Analyses of phylogenetics and evolution in R language. Bioinformatics. 2004;20: 289–290. doi: 10.1093/bioinformatics/btg412 14734327 10.1093/bioinformatics/btg412

[49] Kembel SW, Cowan PD, Helmus MR, Cornwell WK, Morlon H, Ackerly DD, et al Picante: R tools for integrating phylogenies and ecology. Bioinformatics. 2010;26: 1463–1464. doi: 10.1093/bioinformatics/btq166 20395285 10.1093/bioinformatics/btq166

[50] Heath TA, Hedtke SM, Hillis D. Taxon sampling and accuracy of phylogenetic analyses. Journal of Systematics and Evolution. 2008;46: 239–257.

[51] Salisbury BA, Kim J. Ancestral state estimation and taxon sampling density. Systematic Biology. 2001;50: 557–564. 12116653

[52] Warton DI, DUURSMA RA, Falster DS, Taskinen S. smatr 3- an R package for estimation and inference about allometric lines. Methods in Ecology and Evolution. 2011;3: 257–259. doi: 10.1111/j.2041-210X.2011.00153.x

