# Supplementary material for: Genome downsizing, physiological novelty, and the global dominance of flowering plants
Source: PLoS Biol. 2018 Jan 11;16(1):e2003706. doi: 10.1371/journal.pbio.2003706 (PMC5764239; doi:10.1371/journal.pbio.2003706)
Supplement: S1 Table — Ds, stomatal density; Dv, vein density; lg, guard cell length. (DOCX) [file pbio.2003706.s001.docx]

Table S1. Trait and phylogenetic generalized least squares (PGLS) regressions for all species and for only the angiosperms. Trait regressions are in the upper triangle and PGLS regressions are in the lower triangle. Values are regression slopes. Asterisks indicate significance level: *P < 0.05; **P < 0.01; ***P < 0.001

| All species |  |  |  |  |  |
| --- | --- | --- | --- | --- | --- |
|  | Genome size | *D_v_* | *l*_g_ | *g_s, max_* | *g_s, op 70_* |
| Genome size |  | -0.45*** | 0.20*** | -0.22*** | -0.48*** |
| *D_v_* | -0.27*** |  | -0.31*** | 0.54*** | 1.05*** |
| *l*_g_ | 0.22*** | -0.19** |  | -0.26* | -1.31*** |
| *g_s, max_* | -0.13** | 0.32** | 0.19 |  | 1.01*** |
| *g_s, op 70_* | -0.27*** | 1.01*** | -0.54** | 0.45** |  |
|  |  |  |  |  |  |
| Angiosperms |  |  |  |  |  |
| Genome size |  | -0.38*** | 0.21*** | -0.18*** | -0.39*** |
| *D_v_* | -0.27*** |  | -0.33*** | 0.47*** | 1.01*** |
| *l*_g_ | 0.22*** | -0.19** |  | -0.13 | -1.18*** |
| *g_s, max_* | -0.14** | 0.30** | 0.19 |  | 0.94*** |
| *g_s, op 70_* | -0.27*** | 0.98*** | -0.54* | 0.43** |  |
