# Supplementary figures and images for: Genome downsizing, physiological novelty, and the global dominance of flowering plants
Source: PLoS Biol. 2018 Jan 11;16(1):e2003706. doi: 10.1371/journal.pbio.2003706 (PMC5764239; doi:10.1371/journal.pbio.2003706)

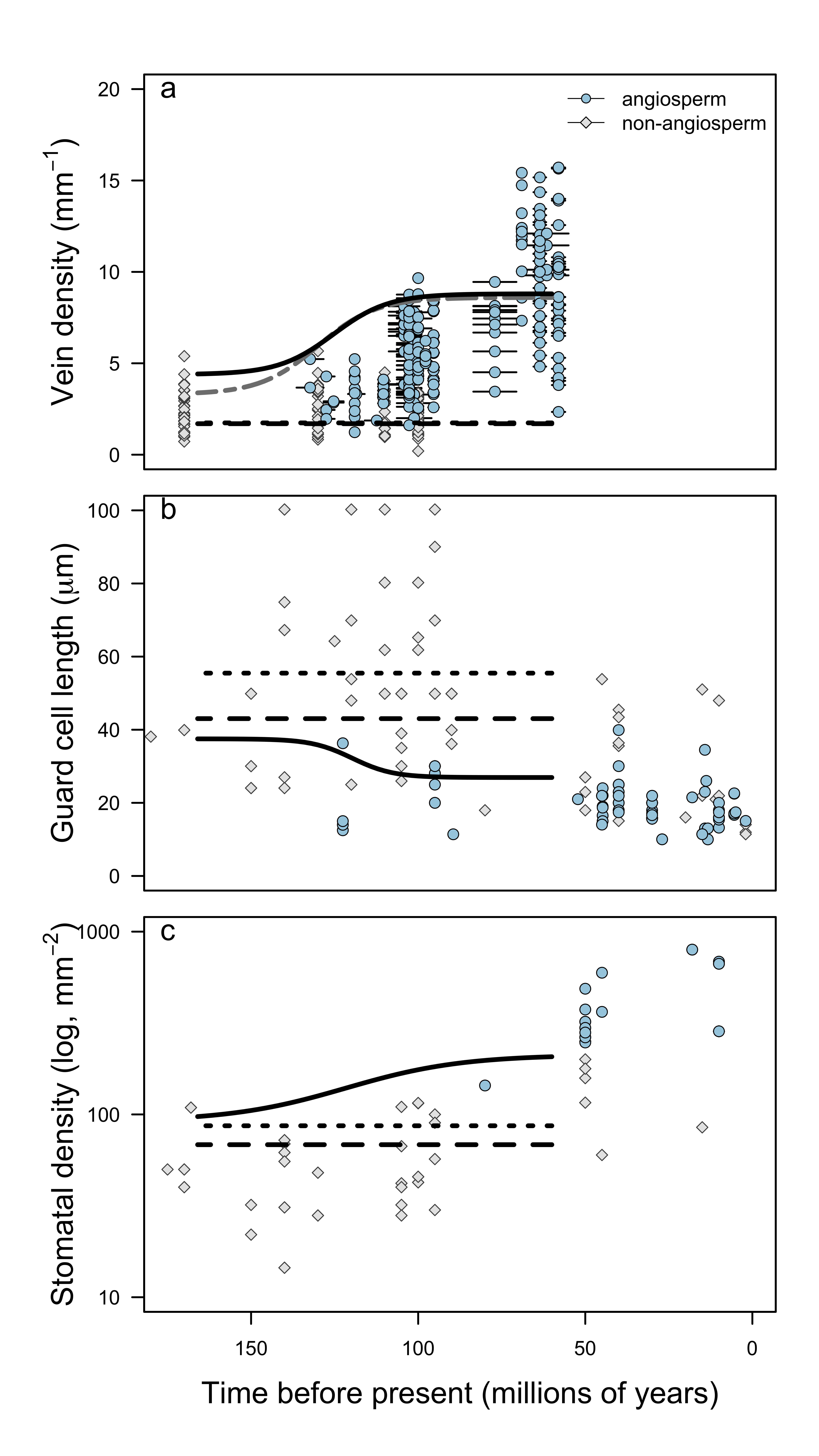

Supplement: S1 Fig — Data can be found in S1 Data. (TIF) [file pbio.2003706.s004.tif]

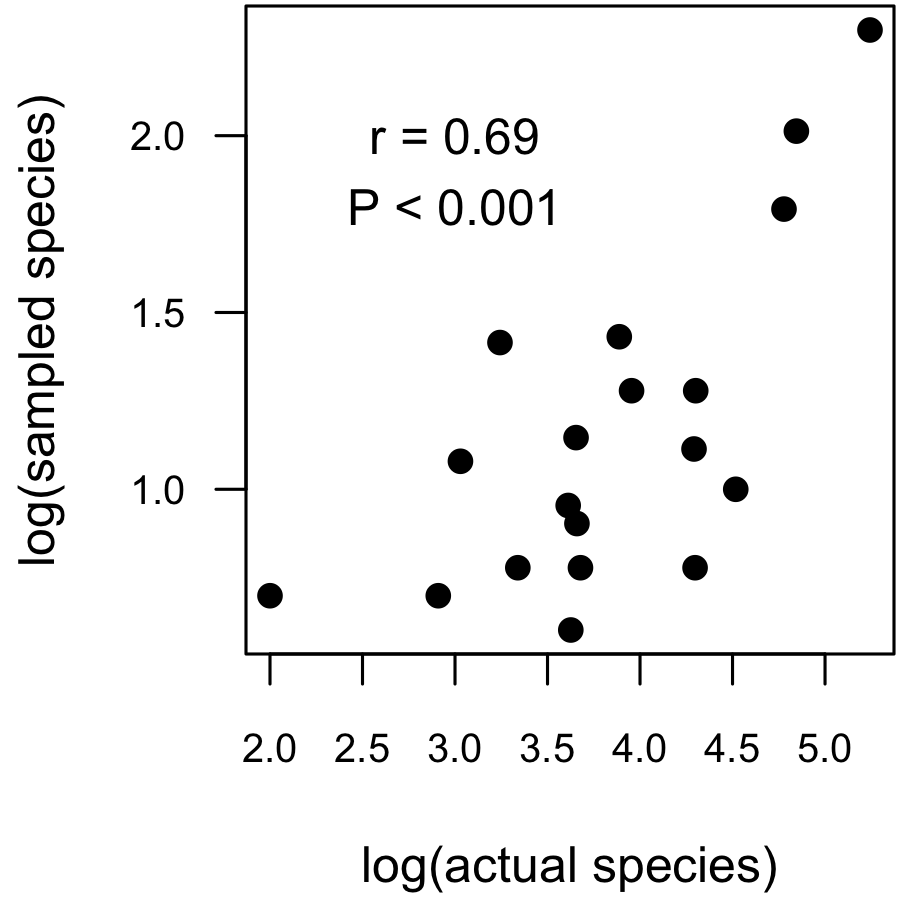

Supplement: S2 Fig — (TIFF) [file pbio.2003706.s005.tiff]

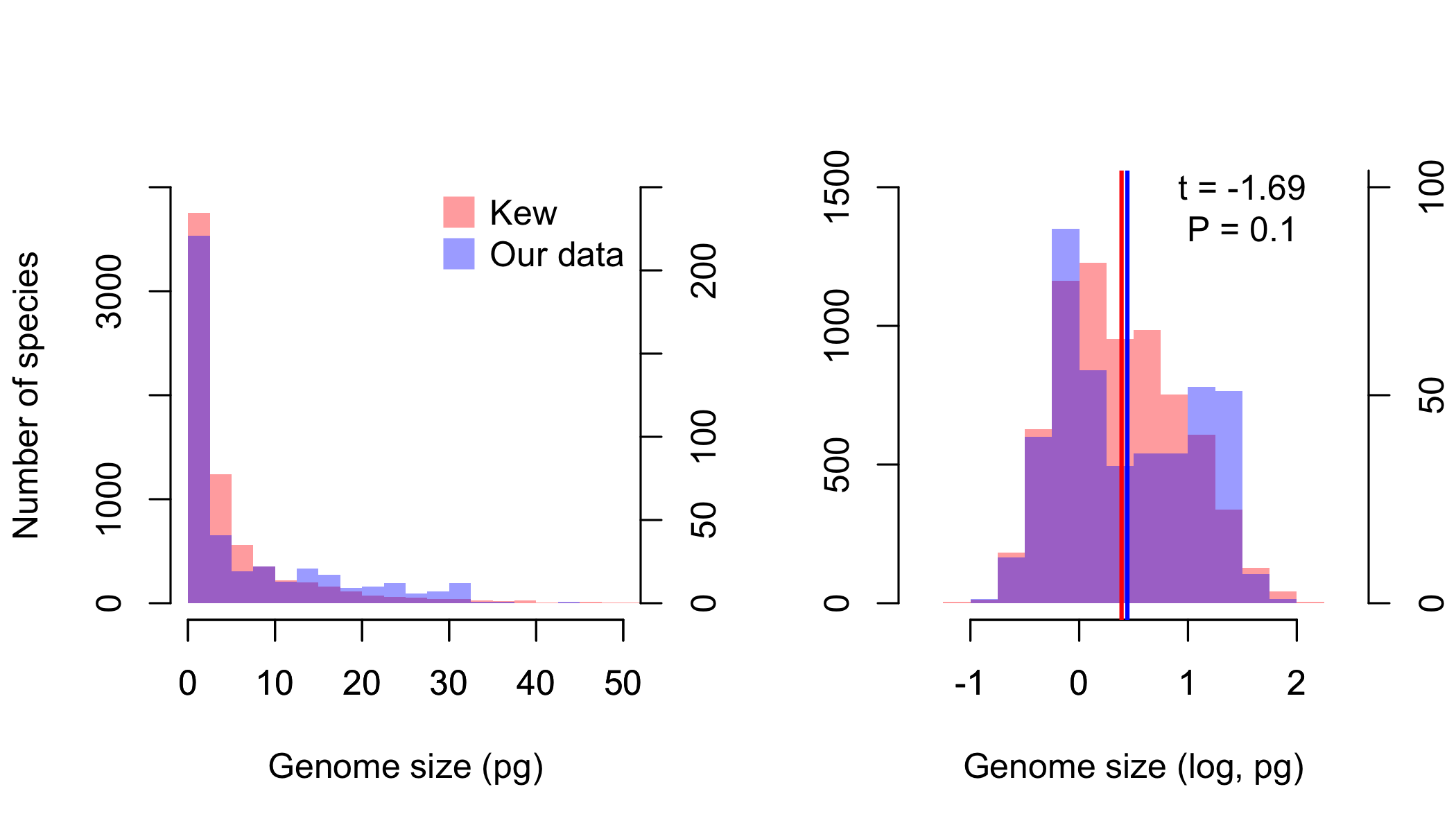

Supplement: S3 Fig — (a) Untransformed distributions and (b) Log-transformed distributions. In both figures, the number of species for the Kew database is on the left axis, and the number of species sampled in this study is on the right axis. (TIFF) [file pbio.2003706.s006.tiff]

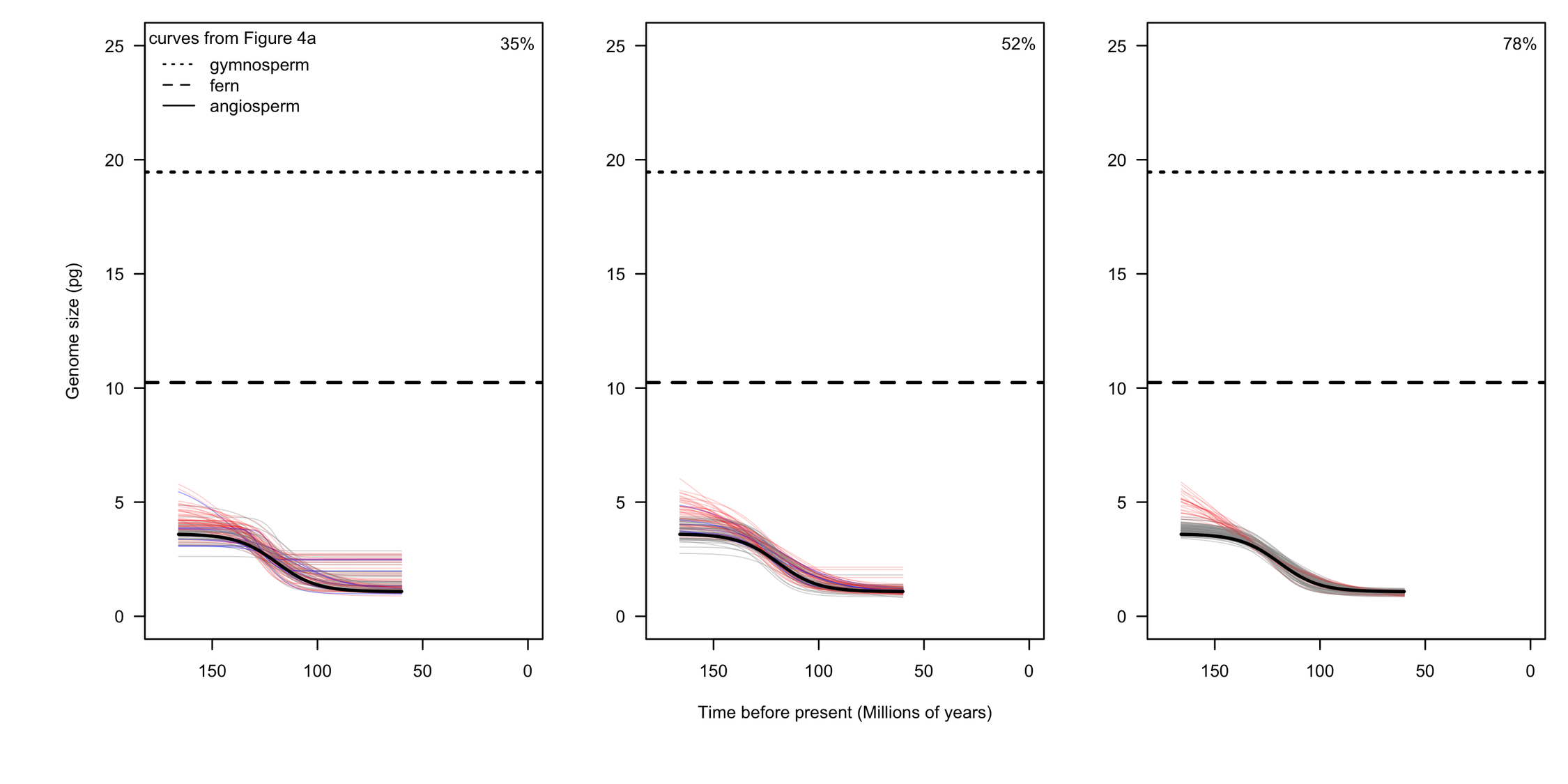

Supplement: S4 Fig — Heavy black lines are the modeled limit from the entire dataset, and the light grey, red, and blue lines are the modeled limits from each of 100 replicate runs at each level of diversity. The modeled limits of genome size for ferns (dashed lines) and gymnosperms (dotted lines) are reported from Fig 4A. (TIFF) [file pbio.2003706.s007.tiff]
